# Knowledge and Attitudes of GPs in Saxony-Anhalt concerning the Psychological Aspects of Bronchial Asthma: A Questionnaire Study

**DOI:** 10.1186/1751-0759-4-23

**Published:** 2010-12-20

**Authors:** Mark G Reed, Daniela Adolf, Katrin Werwick, Marcus Herrmann

**Affiliations:** 1 Otto-von-Guericke-Universität Magdeburg, Leipziger Straße 44, 39120 Magdeburg, Sachsen-Anhalt, Germany

## Abstract

Bronchial Asthma is a worldwide condition with particularly high prevalence in first world countries. The reasons are multifactorial but a neglected area is the psychological domain. It is well known that heavy emotions can trigger attacks and that depression negatively affects treatment outcomes. It is also known that personality type has a greater effect on disease prevalence than in many other conditions. However, many potential psychological treatments are hardly considered, neither in treatment guidelines nor in reviews by asthma specialists. Moreover, there is very little research concerning the beliefs and practices of doctors regarding psychological treatments. Using a questionnaire survey we ascertained that local GPs in Saxony-Anhalt have reasonably good knowledge about the psychological elements of asthma; a third consider it to be some of the influence (20-40% aetiology) and a further third consider it to be even more important than that (at least 40% total aetiology). Our GPs use psychosomatic counseling sometimes or usually in the areas of sport and smoking (circa 85% GPs), although less so regarding breathing techniques and relaxation (c40% usually or sometimes do this) However despite this knowledge they refer to the relevant clinicians very rarely (98% sometimes, usually or always refer to a respiratory physician compared with only 11% referring for psychological help).

## Introduction

Bronchial Asthma is a chronic condition whose worldwide incidence continues to rise unabated, both in richer countries and those with emerging economies [1]. Its pathophysiology is better understood than ever before and current treatments are more effective than ever. However, the costs incurred by the disease continue to increase and young people continue to die from it. One area of aetiology that is often neglected in the Western World is the psychological element of asthma; by neglecting this area, potential treatments are also neglected [2-4]. Early in the twentieth century many prominent thinkers had overemphasized the psychological basis of disease; such work is often considered discredited nowadays, but we should not forget how important psychosocial factors are in all diseases. Indeed in 1965 Michael Hirt edited a book [5] with the aim "to stimulate research on the psychosocial implications of asthma, which will meet the vigorous methodological conditions possible in the laboratory setting". A review in JAMA at the time stated "It is extremely doubtful that this publication will achieve such a goal". This second prophecy has proven correct; we can find little such good quality research and no data at all about the beliefs and practices of doctors in this area. This contrasts with other treatment modalities, for example Vollmer et al's paper in 1997 [6] which compared allergy specialists and general physicians pharmacological treatments of asthma.

Doctors in the English speaking world are particularly reticent about psychosomatic medicine (such as chronic fatigue or irritable bowel syndrome [[7] and [8]]). There are many reasons for this, but perhaps a dominant one is the culture of political correctness; doctors fear offending their patients by implying their illness is "all in the mind"; we may mistakenly think that our patients themselves are convinced their illnesses are entirely physical, whereas many studies have shown that patients actually do incorporate psychological explanations into their health belief models [9]. We worry they will think we are saying it is their fault, that they should just pull themselves together. Other countries do not feel this reticence; for example, in Germany there are institutes of psychosomatic medicine to which people can be referred by their GP. How would this be perceived in Britain? In America, psychosomatic medicine does have a more respectable profile as illustrated in a review article by Weiner [10]; however it is still somewhat neglected there as emphasised in a JAMA editorial 1999 by Spiegel [3] who writes about how social factors are strongly linked to physical disease outcomes, although their influence is often ignored.

**Figure 1:**
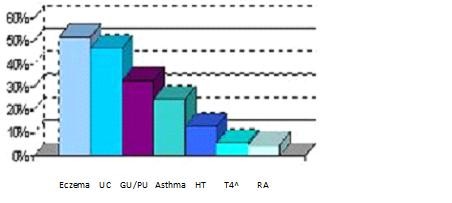
**Percentage of GPs who see psychological factors as pre-dominant in the development of the following**. A graph of GPs beliefs about the psychological influence on a number of common diseases.

In 1950 Alexander published a work that described seven conditions thought particularly to have a psychosomatic aspect [11]. These were reverently referred to as the "Holy Seven" in the following years [12]. They were: gastric ulceration, ulcerative colitis, bronchial asthma, essential hypertension, eczema, hyperthyroidism and rheumatoid arthritis (see Figure 1 in results). Modern doctors recognise a psychological element to all illness and hence Alexander's choice of these seven diseases is now considered outmoded [13]. In these days of holistic medicine it is wise to consider the psychosocial domain in most medical conditions, although the degree to which this affects disease expression varies. Indeed, there is much evidence that psychosomatic factors are particularly important in asthma.

In an otherwise very thorough review of asthma control in the BMJ in 2006 John Rees [14] only mentions psychological factors twice:

"Factors that are difficult or impossible to alter: Psychological problems"

as well as:

"In the future it is likely that appropriate interventions such as cognitive behavioural therapy will be available to target adherence in particular patients, although as yet evidence is lacking to support any particular intervention."

However, the evidence deserves more than this. He does not mention at all the wealth of research that shows positive outcomes for children if the psychosocial domain is influenced. As long ago as 1993 Bryan Lask [4] was lamenting the lack of attention given in British Thoracic Society (BTS) guidelines to the psychological causes and management of asthma; for example, parental counseling [15], and family therapy [16] have proven benefits but are not mentioned. In adults cognitive behavioural therapy [17] has been shown to have beneficial effects. This situation has not changed in the most recent BTS guidelines [18], where the only mention of anything psychological was the following, found under 'complementary and alternative medicines':

"In difficult childhood asthma, there may be a role for family therapy as an adjunct to pharmacotherapy".

The situation is slightly better with the German asthma guidelines [19]: there is a section for "Non-medical Measures" (Nichtmedikamentöse Maßnahmen) in which it is emphasized that family relationships are important to success in treating children, and that psychosocial problems in all age-groups are common. Moreover it is mentioned that psychosocial factors will play a role in the development and maintenance of asthma and support should be tailored accordingly. However there is no advice about who to refer, when, or to what specific psychosocial therapeutic intervention.

It has long been well-known that severe asthma attacks can be triggered or worsened by heavy emotions. Groen and Decker [20] wrote in 1956 about "reproducible psychogenic asthma attacks" in a fascinating paper that would not pass submission to a modern ethical committee. Periodically such work gets revisited (in less dangerous ways) such as Ritz and Steptoe [21] who demonstrated how laboratory induction of emotions in adults can bring about attacks. Strunk et al [22] conducted a retrospective case control study of children who died of asthma (average age 13), following discharge from hospital in the late 1970s. They examined 57 physiological and psychological variables gathered during admission and found eight that had prognostic significance. Four were psychological in nature: concurrent depression, self-care inappropriate to age, parental conflict with medical staff and disregard of asthma symptoms by patients or parents.

There is a growing body of work examining psychosocial influences on childhood asthma. The effect of emotions was further examined in a 2009 paper by Suglia et al [23]. They found that, all other factors being equal, children of mothers who experienced chronic intimate partner violence had over twice the risk of developing asthma. This effect was reduced in children who experienced a high level of mother-child activities and further reduced if the child had a higher number of toys (after taking into account obvious confounders such as income and smoking).

So, it is known then, that psychological factors can affect disease expression. Indeed depressive co-morbidity has been shown to be commonly associated with asthma [[2] and [4]]; but is this cause or effect? This hypothesis was tested by Bender et al [24] in with respect to mild or moderate asthma in children. They were surprised to find that whilst the presence or degree of asthma affected their young lives in various ways, the psychological functioning of the children was not linked to their asthma. In fact it was the Family's Adaptation (measured by the "Impact on Family Scale") that was related to the coping skills of the children in question. Perhaps this is not such a surprise to experienced GPs who know that children cope very well with many physical problems and suffer most when there are background family difficulties. It has also been observed in a fascinating study from China [25] that the three basic temperament types affect asthma expression differently: feisty ("difficult") children were found to have asthma three times more than average whereas flexible ("easy") children had it only half as often. Indeed the authors began their abstract by saying "Asthma is considered as a typical psychosomatic disease."

Many studies have demonstrated that adults with depression and anxiety perceive themselves as having more severe physical disease, and cope less well with their illnesses. Rimington et al [26] showed this clearly for asthma: adult patients who are more depressed or anxious scored worse on symptom scores; it was found such symptom scores were much less strongly related to actual lung function than to the results of the Hospital and Anxiety Depression scale. Furthermore they suffer more from poor control [27]. Indeed, Kolbe et al [28] found a higher incidence of anxiety, depression and life events in asthmatics of all ages admitted with severe attacks.

Of equal importance is the question of whether psychological therapies can improve the outcome in physical disease? A succinct study by Smyth et al in JAMA 1999 (prompting Spiegel's editorial) demonstrated that simply writing about stressful experiences could improve lung function at 4 months follow-up in adult patients with mild to moderate asthma; there was in fact a relative improvement of nearly 20% in FEV1; P value < 0.001 [29]; this was a randomized controlled trial, with the control group writing about neutral topics.

There is also evidence that behavioural techniques can help asthma. Colland [30] demonstrated that a self-management program was effective in reducing asthma morbidity in children, and other centres have extended this work [[31] and [32]]. This has been further extended by Grover et al in 2007 [17] who demonstrated in a randomised controlled trial that CBT was superior to self-management programs in adults with asthma. On a different theme Huntley et al [33] reviewed all relaxation techniques and their efficacy on asthma; they concluded most studies were of poor quality but that there was some evidence that muscular relaxation improved lung function.

So then, we know that psychological factors affect development, severity, and mortality in asthma. Moreover psychological therapies can improve disease outcome. The research above demonstrates that this applies to children, adolescents and adults. However, it is one thing for doctors to be aware of aetiology and treatment; it is another matter for them to act accordingly. Hence, we decided to investigate the knowledge and behaviour of our local GP population. In order for this to be done in as simple a manner as possible we sent our GP colleagues a questionnaire looking at their health beliefs and querying their referral patterns. We hypothesised that local GPs would probably be aware of the psychosocial links but that they were often likely not to refer or treat accordingly. We examined this hypothesis in several areas of aetiology and treatment (see methods).

In the interests of space we have not included the questionnaire in this paper but we are happy to email it to any interested parties.

## Method

424 GPs in Saxony-Anhalt, a state in former East Germany, were sent a locally-constructed questionnaire (90% by fax, 10% by post, reply possible either way) concerning the psychosomatic aspects of general practice. This is just over a quarter of GPs in the state, a number which was chosen in order to achieve a satisfactory cross-section whilst not demanding an unnecessarily excessive analytic workload. The practices were chosen at random and an analysis of respondents was done to ascertain how similar they were to the figures for the state as a whole.

Specifically questioned were:

• opinions about the aetiology of asthma

• their referral patterns

• treatments advised

• awareness of psychosomatic links with asthma

• their patients' concordance.

There were two further elements to the questionnaire - a five point scale response for some of the questions and also a free text response area.

The GPs were asked for the following demographical variables (each category was divided into 2 parts to simplify analysis):

• region (town/country)

• gender

• time working in general practice (before/after 1992)

• patients per quarter (</> 1000)

• additional psychosomatic training (yes/no).

128 questionnaires were returned, of which 6 were unusable, a response rate of 29%. The demographic breakdown is shown in Table 1 and Figure 1.

**Table 1:** Demographic breakdown of questionnaires

Properties:	Total	Place		Year		Patients	
**Figures in %**		**Town**	**Country**	**< 1992**	**> 1992**	**< 1000**	**> 1000**

Male Drs (SA)	39	55	45	44	56	28	72

Male Drs (Q)	40	55	45	50	50	24	76

Female Drs (SA)	61	41	59	47	53	34	66

Female Drs (Q)	60	21	79	70	30	36	64

Q - Questionnaire Respondents (total 128 replies)

SA - Saxony-Anhalt Statistics (total 1461 GPs)

We analysed the data using SPSS 15 to look at correlations and frequencies. For testing differences of frequencies we used Fisher's exact test. Focussing on the main result, we performed a Bowker test on combined frequencies. To test whether the demographical variables influenced the awareness of psychological links, a multivariate ANOVA was done. A P-value less than or equal to 0.05 is considered as indicating statistical significance. The Original questionnaire was in German. A translation is available on request.

## Results

Analysis of the population of questionnaire respondents (Q) showed that it did not represent exactly the same population as doctors in Saxony-Anhalt in general (SA). Notably, more female doctors who qualified before 1992, and who practised in the country replied than would be expected statistically.

It can be seen that more women practice as GPs in Saxony-Anhalt (61%) although a slightly greater proportion have less than a thousand patients (34% v 28%). These data show that more of our female respondents practice in the country and qualified before 1992 than would be expected from the Saxony-Anhalt statistics; these differences are statistically significant with P = 0.004 and P = 0.002 respectively.

These are the "Holy Seven" psychosomatic diseases written about by Alexander in 1950. Notably about half of the GPs consider eczema and ulcerative colitis (UC) to have a mainly psychological causality. One third consider this to be so for gastric or peptic ulcers (GU/PU), whereas very few think this for hypertension, hyperthyroidism and rheumatoid arthritis. About a quarter think the psychological element is pre-dominant in asthma.

**Figure 2:**
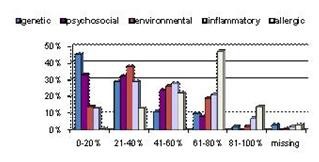
**Weighted importance by GPs of 5 aetiological factors in Asthma**. Bar chart showing GPs beliefs about the relative importance of various causal factors in asthma.

In Figure 2, the Y-axis Percentage indicates the percent of GPs who weigh that factor with the corresponding degree of importance (X-axis). For example 45% of GPs consider that genetic factors have only a 0-20% bearing on asthma aetiology, whereas nearly half the respondents thought that allergic factors were responsible for 61-80% of the aetiology of asthma.

The total weightings for all 5 factors add up to 100% for each GP.

**Figure 3:**
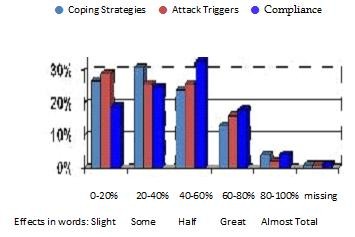
**GPs' beliefs that psychological factors affect the following areas of asthma**. Bar chart showing GPs opinions on how much psychology affects compliance, triggers and coping.

A quarter think that psychosocial factors have 41-60% of the influence which tallies well with Figure 3.

The Y-axis Percentage indicates the proportion of GPs who consider psychological factors to have the corresponding degree of importance on the X-axis.

Here we can see that GPs consider that psychological factors particularly affect compliance (33% think it is worth about half the total influences on compliance, 17% think it is most of the influence, 4% almost total); this contrasts with 25% and 24% of GPs thinking it had a 41-60% bearing upon attack triggers and coping strategies respectively.

**Figure 4:**
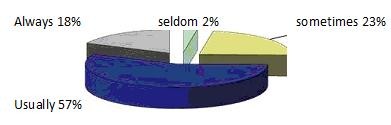
**Percentage of GPs who refer to specialist care (Respiratory or General Physician)**. Pie chart showing how often GPs refer patients with asthma to a respiratory or general physician

**Figure 5:**
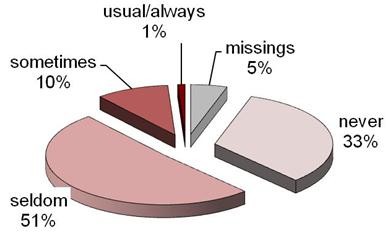
**Percentage of GPs who refer Patients for Psychological Therapy**. Pie Chart showing how often GPs' refer patients with asthma to psychology.

Figure 4 demonstrates that 75% of Saxony-Anhalt GPs always or normally refer to secondary care. This contrasts with 33% never, and 55% seldom referring for psychological care, as seen in Figure 5:

Of the 11% above, who always or sometimes refer patients for psychological therapy this was further broken down into those with or without an additional psychological qualification. We found 0% of those without such a qualification sometimes or always made such referrals, whereas 18% with such a qualification did so.

In comparing whether the belief that psychological factors affect asthma (Figure 3) is in contrast to the percentage of GPs who refer patients for psychological therapy, above we combined the three areas in median and mean values respectively and tested for symmetry. Our results showed P < 0.001 in both cases. This means that although there are GPs who think that psychological factors affect many areas of asthma, they do not then refer their patients for psychological therapy.

We also compared the awareness of psychological aetiology (Figure 1) with the percentage of GPs who refer patients for psychological therapy (Figure 5). Here, also a P-Value < 0.001 for median and mean, respectively, again indicates that even when GPs are aware of psychological links, they do not refer their patients for psychological therapy.

**Figure 6:**
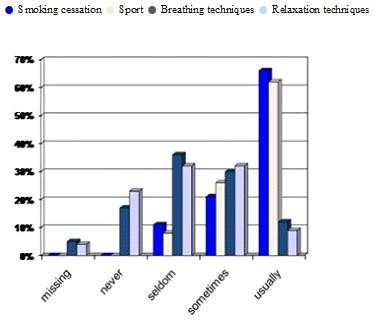
**Percentage of GPs who advise the following**. Bar Chart showing how often GPs' discuss various psychological treatments with their patients.

Figure 6 shows that most GPs sometimes or usually advise their patients concerning smoking cessation and the benefits of sport. Moreover breathing therapy, such as the Buteyko technique [33] and relaxation techniques [34] were discussed sometimes or usually by over 40% of GPs.

## Discussion

Questionnaire studies can often suffer from response bias. Our population data were not the same as the general data characteristics for the entirety of the state of Saxony-Anhalt (see Table 1 and Figure 1). Notably more of our female respondents worked in the countryside and had been longer established in their practices (before 1992). Explaining why is never easy. Perhaps more of these women had children who had attended the new Otto Von Guericke University of Magdeburg in recent years, and were grateful for the new opportunities it provided? This may have made them more well-disposed to returning research questionnaires. The population of male respondents (Q) was statistically the same as that of the general male doctor population in Saxony Anhalt, however.

Figure 2 illustrates the degree to which GPs in Sachsen-Anhalt believe psychological factors affect asthma. Roughly a third think it has little overall impact (0-20% total influence); however a further third think the influence is quite marked (20-40% total aetiology); a quarter think psychosomatic elements explain about half the causation in asthma, and nearly 10% think it actually accounts for most of the underlying aetiology. This demonstrates a clear knowledge of the importance of psychological factors. Despite this, 84% of GPs seldom or never referred patients for specific psychological therapy. This was broken down above into those with or without further qualifications in psychosomatic medicine, although the difference between them was not statistically significant. Many GPs in our locality did, however, demonstrate their knowledge of the importance of the psychological domain by some of their other treatment behaviours, as demonstrated in Figure 6.

We further investigated whether the five demographical GP variables mentioned above had an influence on the awareness of psychological links; we found that female (by trend) and younger doctors were more open to such a link, a difference which was statistically significant for the time working in general practice with P = 0.024. An interesting comparison is drawn with the views doctors have about any psychosomatic link with Alexander's six other diseases (Figure 2); our GPs consider ulcerative colitis, gastric/peptic ulcers and eczema to have more of a psychosomatic element than asthma [10].

Figure 4 illustrates that local GPs normally or always referred their patients for further care from a respiratory physician in 75% of cases. To British doctors this will probably seem like a high figure and it demonstrates that German GPs have more of a gatekeeper role than their British counterparts these days. In Britain the majority of asthmatic children are managed in general practice. This is recommended by the BTS Guideline on the management of asthma [18]; listed within it is a table with "suggested reasons for referral to a respiratory physician", which include brittle asthma, occupational asthma, those on high doses of steroids and those with severe symptoms. Indeed in the UK it is recognised to be good care for GPs to manage "normal" asthma and to leave specialists to deal with the difficult cases [35].

The statistical analysis of Figure 5 is the most important part of our study as it demonstrates the lack of a link between knowledge and action, which is surely a fundamental of practice. Despite understanding that psychological factors impact on a variety of aspects of asthma, GPs do not routinely refer their patients for psychology therapy.

## Comparison with Existing Literature

This study was stimulated in large part by Woeller's 2009 paper from the Clinic for Psychosomatic Medicine, Rhein-Klinik, Bad Honnef [2]; he reviewed and summarised the current understanding of the psychological aspects of asthma, with respect to aetiology, co-morbidity and compliance. We built on this with our questionnaire study. We have not managed to find any other studies examining GP beliefs and referral patterns concerning the psychological elements of asthma. Moreover, despite the wealth of evidence concerning psychological factors for other diseases, there seems to be a paucity of research concerning the beliefs and practices of primary care doctors with respect to psychological therapies in the front line of patient care.

There are some studies that have examined the psychological treatments of respiratory disease emphasising how it is widely recognised that lung disease creates many of the same symptoms as psychosomatic illness, thus making it potentially very amenable to psychosomatic treatments. An example includes a study from Spain [36] that uses psychological mechanisms to help with patient's fatigue, panic, and perceptions about disease and quality of life.

There are few other studies that examine the psychosomatic element of other diseases. An interesting example is a German study [37] that examined the degree of childhood stress in sufferers of ulcerative colitis, one of Alexander's original Holy Seven. Our GPs considered that the psychosomatic component was about 50%, but this study showed that childhood and adolescent stress was of normal levels in sufferers of subsequent UC, undermining the suggestion that psychological factors are an important part of its aetiology.

Conversely, the disease mostly considered to be almost completely non-psychosomatic by our GPs (rheumatoid arthritis) has been shown to have a psychological element in a Japanese study [38] that demonstrated that circulating neuropeptides (which can have a local effect on arthritis) were greater in subjects who scored higher on questionnaire tests of psychological complaints such as anxiety.

## Strengths and Limitations of the Study

Clearly, the numbers of respondents was lower than we would have liked. GPs with sympathy for psychosomatic medicine might have preferentially responded. It would be useful to know the actual proportion of these doctors in practice.

The simplicity of the questionnaire meant that the questionnaire should not have taken more than 5 minutes to fill in (see example in methods); this necessarily reduced the quality of some of the responses, as well as the amount of information that could be gained. For example the answers were not differentiated with respect to the age of the patients, which would have been interesting, but increased the work of filling in the data (and probably reduced the response rate).

## Implications for Future Research and Clinical Practice

This study looked at the beliefs and behaviours of GPs in our local vicinity. These are obviously linked; however, given the beliefs in the value of the psychological domain by our GPs, it was surprising that appropriate referrals were so few as a result. Why do most GPs not refer for psychological treatments when they appear well aware that this can help? Is it because GPs expect the respiratory department to do this instead, given the high rate of respiratory referrals? The reasons behind this should be investigated. Or is it because they engage in psychological therapy themselves? This is surely unlikely given the short time most GPs have with their patients. The answer might be because they feel psychological treatments are limited to smoking, education and self-help, in which areas our GPs do engage. Given the evidence for efficacy it would certainly seem wise for GPs to use psychological techniques to improve asthma outcomes. At the very least they should make their patients aware of the link as knowledge is the first step in any potential change. Future research might prove fruitful in examining why GPs seem reluctant to act on their beliefs in this domain. The question of the proportion of patients who would benefit from an actual specialist psychosomatic referral is one which might benefit from a study being done on a single practice's population of asthmatics for example.

With respect to concordance our GPs felt that nearly 90% of patients "usually" or "sometimes" had problems with this. This is useful information for drug delivery developers in pharmaceuticals but also demonstrates that there is much work to be done on an individual level helping patients cope with their medication regimes. Behavioural techniques, education and psychotherapeutic input are all underutilised in this area.

Although our GPs demonstrated that they both use psychological treatments scarcely and refer little for further treatment it would be interesting to note if there was an age discrepancy. That is, do they consider it is more useful to use psychology for children or adults?

To a degree it is unsurprising, given that psychological factors are scarcely mentioned even in recent BTS guidelines [18], that most GPs engage in a limited manner with the psychological domain in asthma, even if, in Germany they are sympathetic to the evidence. We would like to see, at the very least, proper guidance incorporated into national guidelines on asthma, in both Great Britain and Germany. This should both increase awareness, but also raise the likelihood that evidence-based treatments will follow.

## Conclusion

It has been known for several decades that there is an important psychological element to asthma. Modern medicine, with its emphasis on the physical domain, tends to ignore this. Psychological factors contribute to both the acute and chronic expressions of the disease and there are a number of psychological interventions that are effective in reducing attacks and improving long-term care. Moreover, psychology has an effect on medication use and concordance. This paper tells us that, in Germany at least, GPs are well aware of the importance of the link, and use it to some degree in their consulting but only a minority refer for further psychological input into their patients' asthma.

## Competing interests

The authors declare that they have no competing interests.

## Authors' contributions

MR presented the data initially at the Integrative Medicine Conference in Berlin 2009 and drafted the manuscript. MH was responsible for the design of the study. KW assisted with the design and coordination as well as carrying out the initial analysis. DA carried out the detailed statistical analysis. All authors read and approved the final manuscript.
